# Novel mapping techniques for ablation of non‐pulmonary vein foci using complex signal identification

**DOI:** 10.1002/joa3.13222

**Published:** 2025-01-27

**Authors:** Hiroyuki Kono, Kenichi Hiroshima, Kengo Korai, Kenji Ando

**Affiliations:** ^1^ Department of Cardiology Kokura Memorial Hospital Kitakyushu Japan

**Keywords:** atrial fibrillation, catheter ablation, fractionated potential, mapping, non‐PV foci

## Abstract

The complex signal identification function of CARTO version 8 enables quantitative evaluation of local potential fractionation. We present a case where this advanced technology successfully identified non‐pulmonary vein foci associated with fractionated potentials during sinus rhythm.

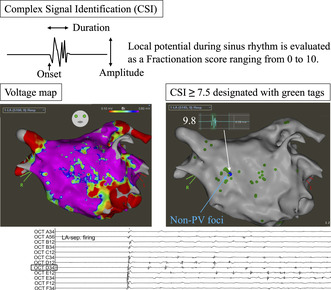

Non‐pulmonary vein (non‐PV) foci are critical ablation targets beyond pulmonary vein isolation (PVI) due to their association with lower recurrence rates when completely eliminated. Conversely, incomplete ablation of these foci often results in high recurrence rates.^1^, ^2^ Despite their importance, the detection and precise mapping of non‐PV foci remain challenging. Using Lumipoint software within the ultra‐high‐density Rhythmia system (Boston Scientific, Marlborough, MA), we identified fractionated signal areas in the atrial muscle (FAAM). FAAM‐guided ablation demonstrated efficacy in detecting non‐PV foci and achieving lower recurrence rates.^3^, ^4^ However, a comparable, well‐correlated mapping strategy for these areas has yet to be established in the CARTO system (Biosense Webster, Diamond Bar, CA).

The complex signal identification (CSI) function in CARTO version 8 is designed to automatically identify and tag complex potentials in the atria. It assigns a fractionation score to each point calculated based on the bipolar or distal unipolar recordings of each potential. Initially developed for detecting fractionated potentials in atrial flutter (AFL), the algorithm utilizes a machine learning model trained on supervised data, incorporating physician‐identified and scored regions of fractionated potential during AFL mapping.

Fractionation scores range from 0 to 10 and are sortable by parameters such as a duration, amplitude, and activation time. Higher scores indicate greater specificity for detecting fractionated potentials but reduced sensitivity. While originally intended for AFL, this approach may also be useful for identifying fractionated signals during sinus rhythm or atrial pacing. We refer to this extended application as Complex Signal Identification for Fractionated signal Area in the Atrial Muscle (C‐FAAM).

A 77 year old female with a 2 year history of paroxysmal atrial fibrillation (PAF) presented with worsening palpitations despite propafenone therapy. Her medical history included gastric cancer, and she was on edoxaban 30 mg and propafenone 300 mg. Laboratory tests revealed NT‐ProBNP 687 pg/mL, creatinine 0.82 mg/dL, and hemoglobin 13.6 g/dL. Echocardiography findings included a left ventricular ejection fraction (LVEF) of 68%, left ventricular diastolic diameter (LVDd) of 38 mm, left atrium diameter (LAD) of 34 mm, and left atrium volume index (LAVI) of 54 mL/min/m^2^.

At admission, the patient was in sinus rhythm. During the procedure, a 10‐pole ring catheter (EP Star Libero, Japan Lifeline Co., Tokyo, Japan) and an Octaray catheter (3‐3‐3 configuration, Biosense Webster, Diamond Bar, CA) were used after transseptal catheterization. Mapping during sinus rhythm (cycle length: 800 ms) revealed a low‐voltage zone (LVZ) on the anterior wall (Figure 1). Pacing from the right superior pulmonary vein (RSPV) reproducibly induced atrial fibrillation (AF).

**FIGURE 1:**
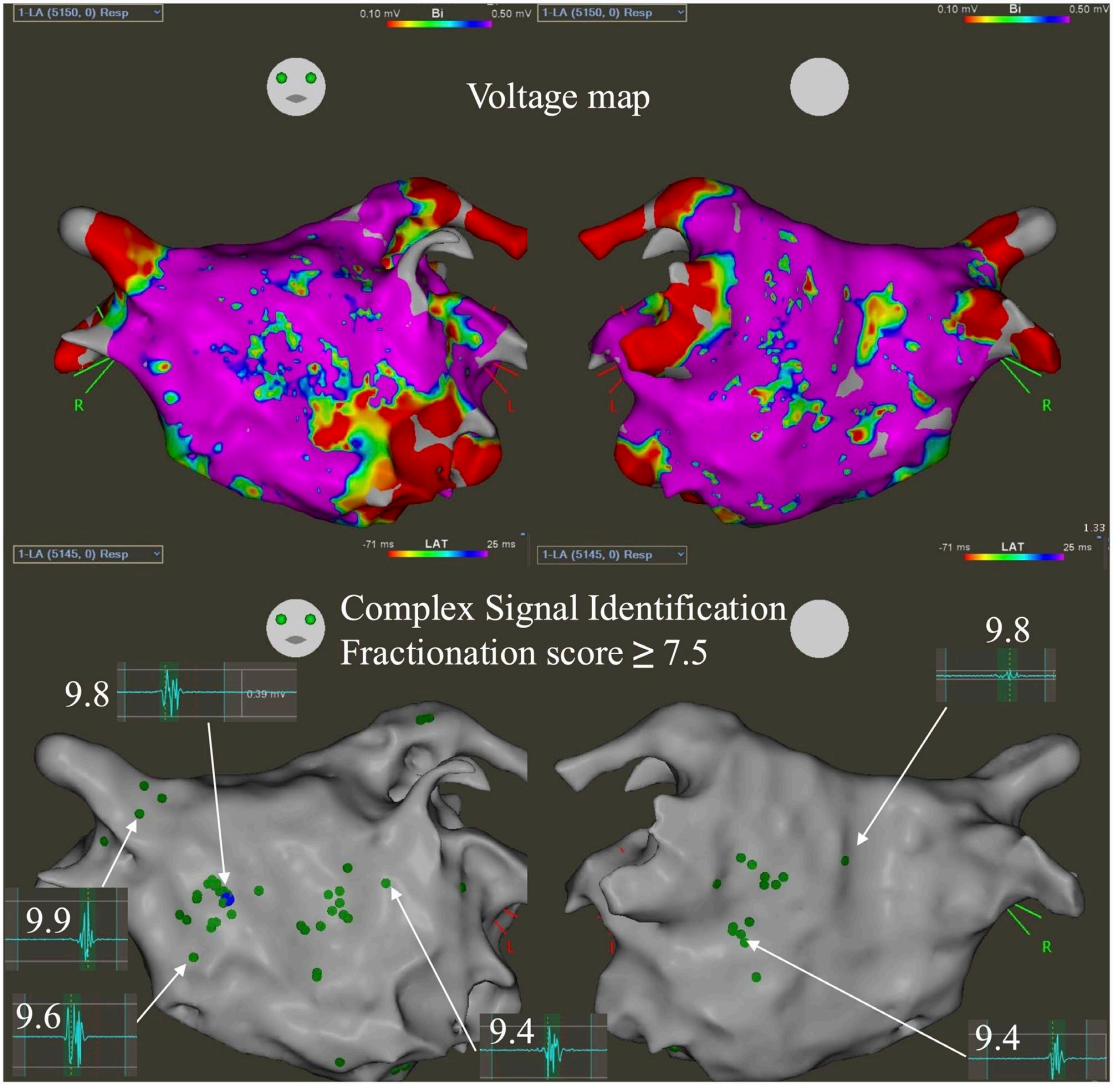
Mapping was performed during sinus rhythm, with complex signal identification (CSI) represented by green tags. CSI distribution varied across voltage zones, transitioning from high to low voltage. The area of atrial fibrillation (AF) firing is indicated by blue tags, corresponding to local potentials exhibiting fractionation, with a CSI score of 9.8.

Pulmonary vein isolation (PVI) and cavotricuspid isthmus (CTI) ablation were performed first. Subsequently, AF was induced with isoproterenol (ISP) and high‐rate burst pacing in the high right atrium (HRA). Following cardioversion, AF was triggered from a non‐PV focus after adenosine triphosphate (ATP) administration. CSI from the bottom of the left common pulmonary vein (LCPV) and the anterior carina of the RSPV extended to the anterior wall.

When a catheter was placed in the area with a high CSI score, the site of earliest activation was identified, matching a CSI score of 9.8 with a duration of 40 ms (Figure 1). Bipolar electrograms from the Libero catheter on the contralateral side showed delayed activation, confirming the site as the earliest activation point (Figure 2). Ablation was performed at this site and in the contralateral region of the right atrium to address the possibility of an intramural lesion (Figure 3).

**FIGURE 2:**
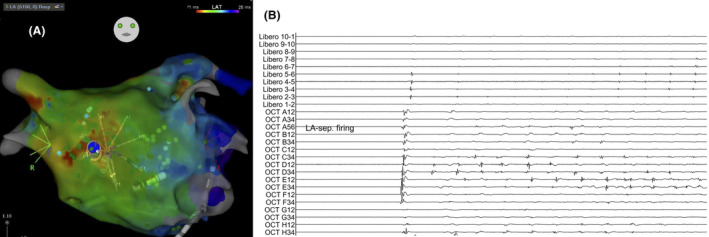
Simultanouesly, the Libero catheter, positioned on the contralateral right atrium septum, showed delayed activation. This confirmed that the blue‐tagged point was a non‐pulmonary vein trigger. (A) Atrial fibrillation was induced in this region, promoting placement of the Octaray catheter at the site. The earliest activation was recorded in electrode D3,4 of the Octaray catheter (B).

**FIGURE 3:**
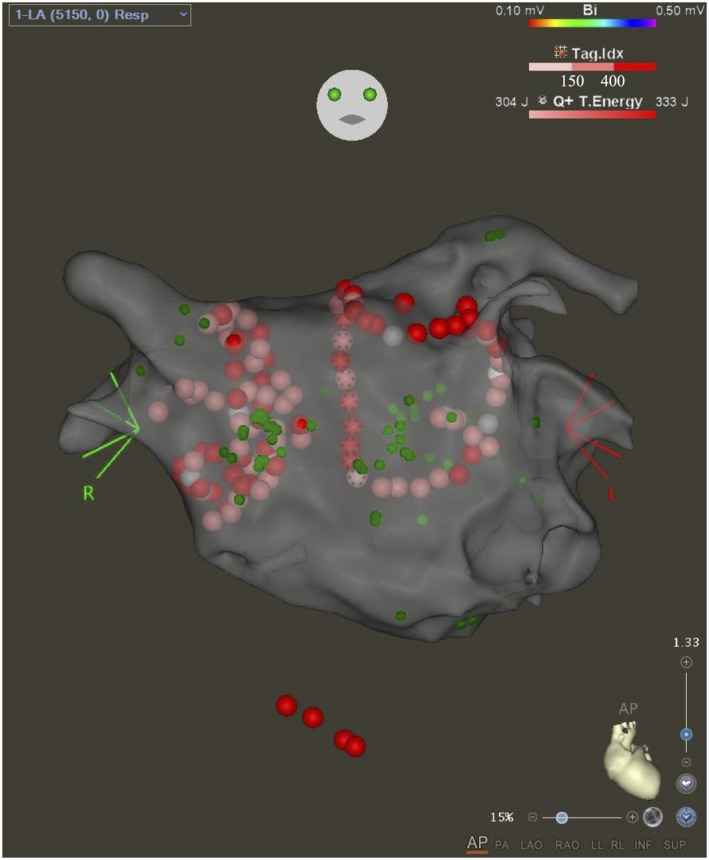
The ablation point is highlighted in this image. Pulmonary vein isolation and cavotricuspid isthmus ablation were performed. The proximal CSI region of the right pulmonary vein and the contralateral right atrium were ablated, and atrial fibrillation could no longer be induced.

Ablation parameters included radiofrequency energy of 25–35 W, with a contact force of 10‐15 g for 10–20 s per application by QDot MICRO catheter (Biosense Webster, Diamond Bar, CA). Posterior wall was ablated in 90 W. The PVI line for the RSPV was slightly extended to include the high CSI area and contralateral right atrium were ablated. AF could no longer be induced.

Postoperatively, the patient was monitored with an ECG for 6 months and maintained a sinus rhythm without the need for antiarrhythmic drugs.

## 1 DISCUSSION

The primary approach to identifying non‐PV foci involves catheter placement to pinpoint the earliest activation site. However, this approach is often challenging, especially when inducibility is poor or when AF originates from a single atrial premature contraction. Non‐PV foci frequently exhibit fractionated potentials, which arise from local conduction delays during sinus rhythm or extrasystole.^5^

CSI‐guided mapping provides an effective strategy for identifying these fractionated potentials. In the presented case, high CSI scores (8.5–10) correlated with regions of earliest activation, enabling precise localization of non‐PV foci. However, CSI's variability, influenced by cycle length and rhythm, remains. A limitation, as the algorithm was originally developed for AFL mapping. Additional validation is needed to optimize CSI settings and improve its reliability in sinus rhythm or atrial pacing.

This study highlights the utility of advanced mapping techniques, such as CSI and FAAM, in guiding ablation for non‐PV foci. Compared to standard mapping, FAAM‐guided ablation has shown superior outcomes, with a 1 year AF freedom rate of 90% versus 75% in non‐FAAM‐guided cases.^4^ The findings suggest that CSI, when appropriately configured, may achieve similar outcomes, though further comparative studies are warranted.

The use of CSI for identifying fractionated signal areas and targeting non‐PV foci in sinus rhythm is a promising approach. However, its clinical application requires further validation in larger cohorts with longer follow‐up durations. Optimizing CSI settings and addressing its inherent variability will be key to improve its utility in AF management.

## FUNDING INFORMATION

This research did not receive any specific grant from funding agencies in the public, commercial, or not‐for‐profit sectors.

## CONFLICT OF INTEREST STATEMENT

All authors have no relevant financial or non‐financial interests to disclose.

## DECLARATION

This study was conducted in accordance with ethical and integrity policies.
